# Conservative Gynecology

**Published:** 1906-01

**Authors:** W. A. Verdier

**Affiliations:** Atlanta, Ga.


ATLANTA

### Journal-Record of Medicine

Successor to Atlanta Medical and Surgical Journal, Established 1855, and Southern Medical Record, Established 1870.

OWNED BY THE ATLANTA MEDICAL JOURNAL CO.

Published Monthly.

VOL. VII. JANUARY, 1906. No. 10.

BERNARD WOLFF, M.D., EDITOR, Nos. 319-20 Prudential.

M. B. HUTCHINS, M.D., BUSINESS MANAGER, 1007-1008 Century Bldg.

E. G. BALLENGER, M.D., ASSOCIATE EDITOR AND ASSISTANT MANAGER.

J. N. LECONTE, M.D., FOREIGN CORRESPONDENT.

### ORIGINAL COMMUNICATIONS.

### CONSERVATIVE GYNECOLOGY.

BY W. A. VERDIER, M.D.

ATLANTA, GA.

The purpose of this paper is to treat generally of gynecological surgery, what should be done under certain circumstances and what should not be done.

This is a broad subject and will of necessity require a rather sweeping paper to serve the purpose for which it is intended.

I may explain, in the beginning, that this subject was prompted by visiting many of the leading surgical institutions of this country and noting the blunders made by men who stand high in the profession, who have had every advantage in training and who have been recognized surgeons for many years.

Therefore, if men of this type are making errors, what of those doing abdominal surgery who have not been so fortunate in securing such excellent training or gaining access to the abundant free clinics of the larger cities, nor possibly the many years of service in this work?

In a day like this when ambition of the young man was never so high, he is apt to do laparotomy, thinking his diagnosis correct, and when he gets on the inside find quite a different condition to deal with, one he failed to review before undertaking this operation and probably a condition which he had never met with before. On the other hand, the older surgeons who do abdominal surgery occasionally and who have not kept up with the progress of this important branch of surgery, for these this paper is particularly written.

As we have no ideal condition to tie to, we will proceed with a case as though she comes to us for examination.

After the history has been given, the next step is to examine the patient thoroughly, note the contour of the abdomen for tumors and surface for eruptions, then our attention will be turned to the inspection of the vulva, venereal sores and eruptions looked for, the color of the parts noted, if any discharge present, its character; the condition of the perineum, whether or not the tenacity of the same has been impaired by parturition, if so, what operation necessary for repair. During the course of inspection, the condition of the anus should be noted, looking out for venereal sores, eruptions, discharge, tenacity of the sphincter muscles, hemorrhoids, fissure and fistulae. After completing inspection we will proceed with

BIMANUAL EXAMINATION.

The first finger of the left hand is inserted in the vagina, first note the tenacity of the vaginal muscles, then as the examining finger reaches the cervix, note the approach. If the pelvic floor is firm and the uterus in proper position the cervix will strike the upper part of the finger and look downward; the finger is then passed on into the cul-de-sac, which would indicate a normal condition of the uterus. However, on the other hand, should you find the floor of the pelvis relaxed and the cervix felt with the tip of the finger looking toward the long axis of the vagina, the anterior wall of the vagina larger than normal and the posterior wall smaller, with the cervix seemingly in the cul-de-sac, you know there is displacement of the uterus. The degree of displacement should be noted and what procedure necessary to rectify it, which is probably a repair of the perineum. With the examining finger pressed against the cervix, the right hand is placed on the abdomen just above the pubes. By this method the uterus can be more thoroughly examined, the size and position of the organ determined. Under normal conditions the uterus is freely movable and unless this condition is found you will suspect adhesions due to previous peritonitis unless tenderness would indicate an active inflammatory condition.

We now pass on to the examination of the appendages. The examining finger passed into the cul-de-sac and the palpating hand moved slightly to the side examined, the tube and ovary can thus be palpated, particular attention being paid to the tubes and broad ligaments. In many cases a small mass here due to tubular pregnancy may be overlooked.

The ovary is not so easily palpated, and in fact not often mapped out at all by the inexperienced, the usual error being made in an effort to reach too high with the examining finger and the palpating hand. This effort hurts the patient and the parts are rendered tense, resulting in failure to map out the ovary and the examination is a complete failure. The correct method is to use little or no force with the examining finger, and the palpating hand, firm but gently placed just above the pubes, a little to the side examined, the ovary is thus felt gliding beneath the palpating hand. Both sides are thoroughly examined in this manner.

With your examination completed you will now make up your diagnosis, and let me beseech you to not talk too much; this is where your patient will be very solicitous as to complete state of affairs. She will expect you to tell her exactly what the trouble is, what the remedy to be is, and the prognosis; but for your sake, the sake of your reputation, don't talk too much. Lose yourself in thought for a few minutes, and leave her questions unanswered, at least until you have weighed the case thoroughly; then should you decide to advise a laparatomy it will be sufficient to say that an operation is necessary, without going into details.

On the other hand, should all the organs be normal as to size and place, but a cellulitis, vaginitis, endometritis, or ovaritis exist, I warn you against the usual flattering prognosis; i. e., "you will soon be well under the douche and tampon treatment, and an operation is not necessary."

I would advise under these conditions that the simpler methods be tried first, and should we fail in this attempt the operation of currettage be resorted to. Your patient may tell you that her family physician has performed this operation several times at her home; however, you should pay no attention to this, for you know this much-abused operation by the average practitioner can not be properly done under such conditions, therefore you should advise curettage before advising laparotomy. Many cases seeming to be mild in the outset, and responding partially to treatment, do not get well from these simpler modes of treatment, and soon they present themselves in exactly the original condition.

In my opinion it is proper here to advise laparotomy. With the present development of surgical technique there is little danger attending this procedure, and in nearly all these cases you will find the cause of the inflammation; sometimes bands of adhesions—the result of a former peritonitis—may be the sole cause. In a large per cent. of cases you will find a degenerative ovary, which is alone responsible for this condition; in such cases ovariotomy should be done.

### OVARIOTOMY—WHEN AND WHEN NOT THIS OPERATION SHOULD BE PERFORMED.

Whether both ovaries should be completely extirpated, or one ovary and a part of the other should be removed, or complete extirpation of one organ, leaving the other, is a question which requires as much study as the treatment of the various forms of malignant diseases. Your decision as to what you will do with these cases resembles emergency surgery more than any other class of elective cases, for it is often you find a condition present after opening the abdomen very different from what you expected; therefore, as the final decision is always made in the midst of an operation, it is of the utmost importance that the care of the ovary is at all times well understood.

The indications for complete removal of the ovaries are:

First. When, from any cause, the uterus and tubes are removed, obliterating the channel of illumination of the menstrual flow; fortunately, in the majority of cases where this is necessary the patient has reached an age approaching the menopause, and those nervous conditions often seen in young women, caused by complete castration, bringing on premature menopause early in life, is not a barrier.

Second. Cystic degeneration of the ovary is another indication for the complete removal of the organ in the majority of cases; fortunately, however, only one ovary is usually affected. Complete extirpation of the cystic ovary being done, leaving the other, should give good results.

Third. Atrophic degeneration is another condition commonly found. Maybe one organ alone is affected; in such condition complete removal of the organ is indicated, leaving the other unmolested.

Again, both ovaries are found undergoing atrophic degeneration. This condition renders your decision more perplexing, the age of the patient being an important factor; the farther down the scale her age may be, the more gravity is added. The nervous condition these women are subject to in later years, after complete castration early in life, is a question well to be considered.

Again, her inability to conceive is another important matter you should bear in mind.

We would conclude, then, that unless it is necessary to remove the uterus, or both tubes, we should never do a complete castration of any woman under thirty-five years of age, and, if possible to avoid it, at any age during the child-bearing period.

The operation *per se* is a simple one—a silk ligature thrown around the blood-vessels at the base of the ovary, tying them off firmly, cutting off all the ovary, leaving a free stump—care taken to see there is no bleeding before closing the abdomen.

When only part of the organ is removed, no ligature is required, the atrophic area is simply cut off.

Some operators have tied off the blood-supply *en masse* and left the ovary in this condition with the idea in view of bringing about gradual menopause. This is only mentioned to be condemned.

REMOVAL OF THE UTERUS.

The indications for removal of the uterus may serve our purpose here best by broadly classifying them as malignant and non-malignant. When operating for malignancy of the uterus or its appendages a complete removal of all the organs of generation should be done, including the cervix-uteri, care being taken to remove all pathological tissue. Unless the operation is thorough it is of no avail. In non-malignant cases, requiring the removal of the uterus, the cervix is left unless actually involved.

The uterus is cut off at its juncture with the cervix, the cut surfaces are folded on each other and sutured. In other words, the cervix should always be left if possible, the atrophy of the vagina is less when it has been left and the use of that organ has not been damaged. However, when necessity demands removal of the cervix, as stated above, there will be shortening and a general atrophy of the vagina.

APPENDECTOMY.

It is becoming very fashionable nowadays with some surgeons to perform this operation in every case where laparotomy is done by the super-pubic route, complimentary, as a kind of "bargain-counter" act. I consider this very unwise, for several reasons:

First: Because it is a general unwritten law that only pathological tissue should be removed. The fact that we have not discovered the function of the appendix does not justify us in treating this member with any less respect.

Second: You subject your patient to the danger of an additional operation unnecessarily and at a time when she can least afford it.

Third: You are not compensated for the service, increasing the mortality of your operative cases, thereby doing yourself and patient an injustice.

LAPAROTOMY.

There are two methods in general use by the gynecologists. The super-pubic median line incision and hysterectomy. The former being the better and more generally used, therefore we will take it up first. The usual method is to cut down on a line with the linia-alba until you are into the abdomen, enlarging your incision to any length necessity may demand. No doubt this will always remain the most reliable route.

However, there is another plan of doing a super-pubic operation which strikes me as being less liable to hernia. A transverse incision is made just on a line of the upper edge of the super-pubic hair, through the skin and superficial fascia, then the muscles are cut through as usual on the line of the linia-alba.

Post-operative the patient will show but little if any scar.

### HYSTERECTOMY.

This is an excellent route for an exploratory incision. All the pelvic organs can be clearly examined. The greatest usefulness of this operation is for drainage and opening pus tubes. The painful scar in the vagina, caused by this operation, the limited space for doing your work, and the pain given your patient, when ovariotomy is done by the hysterectomy clamps, for forty-eight hours after operating will always stand as a barrier to this route.

### ABDOMINAL SUTURE.

As to the different methods of suturing the abdomen, I think the larger suture is the best. As to selection of material, would suggest the following classification: Catgut for peritoneum, kangaroo tendon for muscle, and silk or silkwormgut for skin.

Some of our best men are still using silk for the muscular coat, with the idea that silk will prevent hernia. This is poor theory. Kangaroo tendon will last as long as the tissue in its grasp and what more could silk do? Buried silk is not readily absorbed, if ever at all. My experience with it has been that buried silk acts as an irritant, causing the tissue around it to undergo cystic degeneration, and every once in a while you will be called to pick them out until they are all removed, which may cover a period of time from six to twelve months.

RESUME.

The object of all surgical procedure is to build and not destroy. This idea holds good in gynecology as well as any other class of surgery. For instance, a neurasthenic, anemic woman, always suffering from her ills, is incapacitated for any service. She dreads coition and the prospect of raising a family is despaired of. This can all be caused by an atrophic ovary, the removal of which will restore her to her former strength, vigor and usefulness. It looks like a bold thing to say the removal of an ovary will improve the sexual appetite, but it will in those individuals who have been rendered incapacitated by diseased ovaries; they regain their usual vigor, sexual appetite returns and if any part of either ovary is left they stand far better chances for bearing.

The painful scar in hysterectomy should be referred to again to emphasize the danger of this operation. Notwithstanding the incision is made in the superior surface of the cul-de-sac, just posterior to the cervix, where the nerve-supply is less than any other part of the vagina, the scar tissue formed there to seal this wound is usually of considerable size, and as scar tissue is of low vitality, being scantily supplied with blood-vessels and no nerves at all, will at times give a sense of pain for which there is no relief.

Complete castration of young women is the cause of another distressing condition I wish to emphasize. The first year after operation your patient is nervous and unfit for anything on account of her premature menopause. When she seems to be fairly well of this nervous condition she apparently improves very rapidly; her weight will probably exceed all previous records and her complexion superb, but when you begin to compliment her on her excellent apparent health, she hails you with a most distressing state of affairs. She tells you that her nerves are in a most wretched condition, even worse than before, and monthly when she should menstruate her distress is unbearable, her head and whole spine feel as though they would burst. There may be effort at vicarious menstruation, bleeding at ears and nose, even sloughing of the tonsils may appear. You would expect from her rosy complexion that her blood was in excellent condition. Examination shows a low blood count which is pathognomonic of anemia, the very opposite of what you expected. You would naturally prescribe your favorite preparation of iron. Finding that gave no results you would resort to another, and so on until the whole list have been tested to your full satisfaction and utter failure to have any influence on blood count.

Some physicians treat these cases systematically, relieving this excruciating monthly pain with bromides, giving as much as 60 grains at a dose and then forced to resort to heroic doses of morphine.

I am sorry to say the etiology of this condition is an error of the operator and the damage done can never be repaired. Palliative treatment is all that can be done. Deplete the system with salines, commencing a few days, if possible, before the expected attack, reducing blood tension. Diuretics should be freely administered; the nervous conditions controlled by such somnifacients as do not depress secretions as chloral, bromides and even hyoscine. Morphine should never be allowed.

By avoiding these danger signals, the gynecologist has a broad field for doing good. The shattered confidence at present as a result of errors of the past will soon disappear, and the many sufferers who are staying away will soon learn that the gynecologist has at last reached that stage of perfection where they can rely on his decision with absolute assurance.

